# Pioglitazone Attenuates Drug-Eluting Stent-Induced Proinflammatory State in Patients by Blocking Ubiquitination of PPAR

**DOI:** 10.1155/2016/7407153

**Published:** 2016-06-14

**Authors:** Zhongxia Wang, Tao Zhang, Lizhe Sun, Ruifeng Li, Yuanyuan Wei, Xiaojuan Fan, Zuyi Yuan, Junhui Liu, Tao Chen

**Affiliations:** ^1^Department of Cardiology, First Affiliated Hospital of Xi'an Jiaotong University, Xi'an 710061, China; ^2^Department of Medicine, Gansu Provincial Hospital of TCM, Lanzhou 730050, China; ^3^Department of Cardiology, Xi'an Central Hospital, Xi'an 710003, China

## Abstract

The inflammatory response after polymer-based drug-eluting stent (DES) placement has recently emerged as a major concern. The biologic roles of peroxisome proliferator-activated receptor-*γ* (PPAR-*γ*) activators thiazolidinedione (TZD) remain controversial in cardiovascular disease. Herein, we investigated the antiinflammatory effects of pioglitazone (PIO) on circulating peripheral blood mononuclear cells (MNCs) in patients after coronary DES implantation.* Methods and Results*. Twenty-eight patients with coronary artery disease and who underwent DES implantations were randomly assigned to pioglitazone (30 mg/d; PIO) or placebo (control; Con) treatment in addition to optimal standard therapy. After 12 weeks of treatment, plasma concentrations of high-sensitivity C-reactive protein (hs-CRP), interleukin-6 (IL-6), tumor necrosis factor-*α* (TNF-*α*), and matrix metalloproteinase-9 (MMP-9) were significantly decreased in PIO group compared to the Con group (*P* = 0.035, 0.011, 0.008, and 0.012, resp.). DES-induced mRNA expressions of IL-6, TNF-*α*, and MMP-9 in circulating MNC were significantly blocked by PIO (*P* = 0.031, 0.012, and 0.007, resp.). In addition, PIO markedly inhibited DES-enhanced NF-*κ*B function and DES-blocked PPAR-*γ* activity. Mechanically, DES induced PPAR-*γ* ubiquitination and degradation in protein level, which can be totally reversed by PIO.* Conclusion*. PIO treatment attenuated DES-induced PPAR loss, NF-*κ*B activation, and proinflammation, indicating that PIO may have a novel direct protective role in modulating proinflammation in DES era.

## 1. Introduction

Polymer-based drug-eluting (everolimus, zotarolimus, sirolimus, paclitaxel, etc.) stents (DES) have been known as a standard treatment for coronary artery diseases (CAD) undergoing percutaneous coronary angioplasties. Although DES has demonstrated efficacy and safety in clinical studies, human pathological data have raised concerns about the long-term healing and the potential for local inflammatory reactions [1, 2]. Also, it has been reported that DES induced hypersensitivity reactions with interacting lymphocytes, macrophages, multinucleated giant cells, and eosinophils and pervasive inflammation throughout the stented arterial segment by autopsy [3]. DES-induced inflammatory reaction has been found as early as 30 days after implantation but progressed in frequency and severity through 90 to 180 days in porcine model [1]. Furthermore, our previous studies found that DES implantation induced specific systematic inflammatory state, as evidenced by the enhanced NF-*κ*B activity, suppressed PPAR-*γ* activity, and elevated plasma inflammatory factors, compared with no-stent implantation or bare metal stent (BMS) implantation [4, 5]. However, how to refine DES-induced proinflammation remains unknown.

In vascular wall and atherosclerosis, PPAR-*γ* is expressed in macrophages, T cells, endothelial cells, and vascular smooth muscle cells [6, 7]. Recent data have shown that synthetic antidiabetic thiazolidinediones (TZDs), which are known as PPAR-*γ* activators, inhibit inflammatory cytokine production by cells of monocyte/macrophage lineage [8]. These activators inhibit gene expression in part by antagonizing the activities of transcription factors such as activator protein-1 (AP-1) and nuclear factor-*κ*B (NF-*κ*B) [9, 10]. Several animal studies have demonstrated the antiatherogenic effects of TZDs [11, 12]. Moreover, pioglitazone (PIO) significantly decreased the occurrence of all-cause mortality, nonfatal myocardial infarction, and stroke in diabetic populations in the Prospective Pioglitazone Clinical Trial in Macrovascular Events (PROactive) [13] and in a meta-analysis study [14]. However, a meta-analysis of rosiglitazone trials in diabetic patients showed that rosiglitazone was associated with increased myocardial infarction and cardiovascular death [15]. In an animal study, PIO was found to increase macrophage apoptosis and plaque necrosis in advanced lesions in LDLr-deficient mice [16]. Another animal study found that pioglitazone could induce excessive hepatic triglyceride accumulation and increase the plasma cholesterol [17].

These conflicting and controversial findings highlight the uncertainty regarding the effectiveness of using TZDs to treat atherothrombotic disease. Whether PIO have atheroprotective effects in DES-treated CAD patients remains unknown. Here, we performed a random single-blind placebo controlled clinical study to investigate whether PIO have anti-inflammatory effects in DES-implanted coronary artery disease patients.

## 2. Methods

### 2.1. Patients and Study Design

All subjects were consecutively recruited from the First Affiliated Hospital of Xi'an Jiaotong University. 28 nondiabetic patients with coronary artery disease (CAD) and who underwent DES implantations were randomized into two groups: placebo group (Con, *n* = 14) and pioglitazone group (PIO, *n* = 14). The nondiabetic state was determined by a negative history of diabetes mellitus, no treatment with antidiabetic drugs, and assessment of fasting blood glucose and oral glucose tolerance test (OGTT). The diagnosis of CAD was in accorded with the WHO definition. The exclusion criteria included clinical evidence of acute inflammation, tumor and rheumatic condition checked by the elevated CRP and ESR, liver and renal diseases, severe heart failure (NYHA class ≥ II), ejection fraction (EF) < 50%, contraindications to treatment with pioglitazone, and patients who were given immunosuppressants.  Table 1 summarized all subjects' demographic data. Study medication (30 mg/d) for 12 weeks was given in addition to optimal standard treatment, including aspirin, clopidogrel, *β* receptor blockers, angiotensin converting enzyme inhibitors (ACEIs) or angiotensin II receptor blockers (ARBs), and statins. We followed up the patients twice after 4 and 12 weeks. Pioglitazone and placebo were provided by Zhongmei Huadong Pharmaceutical Co., Ltd. (Hangzhou, China).

We obtained each patient's medical and family history and general information such as smoking and drinking by medical history interview. This study complies with the Declaration of Helsinki, and the research protocol has been approved by the Ethics Committee of Xi'an Jiaotong University. The informed consents were obtained from the subjects.

### 2.2. Mononuclear Cell (MNC) Isolation

Peripheral blood MNC samples before medication and at the end of the 12th week after DES stent implantation were collected. Peripheral blood MNC samples were isolated by Ficoll standard density gradient centrifugation. The upper layer containing MNC was harvested and washed with Hanks' balanced salt solution and then with PBS.

### 2.3. Plasma Concentrations of Proinflammatory Cytokines

The concentrations of plasma interleukin-6 (IL-6), tumor necrosis factor-*α* (TNF-*α*), and matrix metalloproteinase-9 (MMP-9) were assayed by ELISA. ELISA was performed by adding 100 *μ*L or 200 *μ*L of each sample to wells in a 96-well plate of a commercially available ELISA Kit (ExCell, Shanghai) according to the manufacturer's instructions. High-sensitivity C-reactive protein (hs-CRP) assays were performed by our hospital's clinical centre laboratory.

### 2.4. Total RNA Isolation and mRNA Detection

Total RNA was extracted from MNC with the RNAfast kit (Fastgen) according to manufacturer's protocol, and real-time reverse transcription-polymerase chain reaction was performed as previous report [4].

### 2.5. NF-*κ*B and PPAR-*γ* DNA Binding Activity

Nuclear proteins were extracted according to the manufacturer's instructions (Pierce). The NF-*κ*B and PPAR-*γ* DNA binding activity were measured with NF-*κ*B p65/p50 and PPAR-*γ* transcription factor assay kit (Abcam) according to the manufacturers' instructions.

### 2.6. Western Blotting

Polyclonal or monoclonal antibodies (Santa Cruz Biotechnology) were used. Densitometry was performed with the Bio-Rad molecular analyst software, and all values were corrected by loading with Gapdh.

### 2.7. Statistical Analysis

Discrete variables were expressed as numbers and percentages and compared by the *χ* ^2^ test. Summary values are expressed as mean ± SE. Skewed data were reported as median (interquartile range). Analysis of the changes from baseline was performed by paired *t*-test. Holm-Sidak two-way repeated-measures ANOVA (TWRMANOVA) method was used for all multiple comparisons between the Con and PIO groups as previous reports [4, 5]. Statistical significance was assumed at the 5%  *α*-error level (*P* < 0.05).

## 3. Results

### 3.1. General Clinical Data

All 28 patients fulfilled the 12 weeks' follow-up without any drug related side effects. The characteristics of patients were shown in Table 1. There were no differences between the PIO and placebo groups with respect to baseline characteristics. After 12 weeks of treatment, there was no significant change in body mass index (BMI), weight, waist to hip ratio, blood pressure (SBP or DBP), fasting glucose and insulin, NT-pro BNP, and EF of the two groups compared to baseline (Table 2). Considering the unchanged BMI, weight, waist to hip ratio, NT-pro BNP, and EF between two groups, there was no evidence of PIO increasing the occurrence of heart failure in the present study. Total cholesterol, triglycerides, and LDL were reduced significantly after medication; however, no disparities of the reduction between two groups were observed.

### 3.2. Pioglitazone Reduced the Plasma Concentrations of Proinflammatory Cytokines

**Figure 1:**
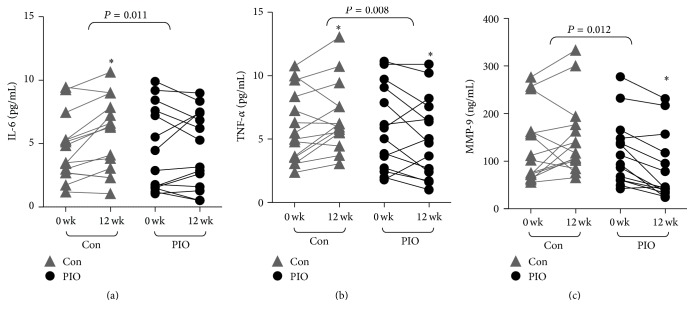
Pioglitazone decreased plasma proinflammatory factors concentrations in patients after coronary DES implantation. The results are presented as raw data. Compared to the control group, PIO significantly reduced plasma IL-6, TNF-*α*, and MMP-9 concentrations by TWRMANOVA (*P* = 0.011, 0.008, 0.002, and 0.012, resp.). ^*∗*^ *P* < 0.05 compared with baseline.

In the control group, plasma concentrations of IL-6, TNF-*α*, and MMP-9 were increased by 138 ± 16%, 136 ± 15%, and 110 ± 7%, respectively, compared with the baseline level at 12 wk (paired *t*-test, *P* = 0.033, 0.041, and 0.142, resp., Figures 1(a)–1(c)), indicating DES-related proinflammatory status. In the PIO treatment group, plasma concentrations of IL-6, TNF-*α*, and MMP-9 were decreased by 89 ± 8%, 64 ± 16%, and 86 ± 6% compared with the baseline level at 12 wk (paired *t*-test, *P* = 0.204, 0.041, and 0.037, resp., Figures 1(a)–1(c)). Compared to the control group, PIO treatment significantly reduced the plasma IL-6, TNF-*α*, and MMP-9 concentrations (TWRMANOVA, *P* = 0.011, 0.008, and 0.012, resp.). Moreover, hs-CRP in both Con and PIO treatment groups were decreased significantly and more drastically in PIO treatment group (TWRMANOVA, *P* = 0.035, Table 2).

### 3.3. Pioglitazone Downregulated the Expressions of Proinflammatory Factors in MNC

**Figure 2:**
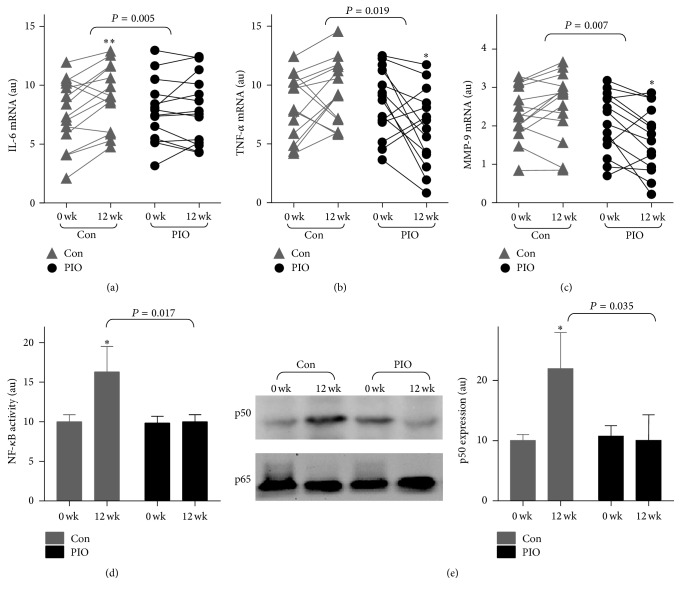
Pioglitazone downregulated the proinflammation in MNC. (a)–(c) Change in mRNA expression of peripheral blood mononuclear cells (MNC). The mRNA levels of IL-6, TNF-*α*, and MMP-9 were detected by quantitative real-time PCR. (d) NF-*κ*B DNA binding activity was detected. (e) The expression of p50 subunit and p65 subunit in MNC. ^*∗*^ *P* < 0.05 and ^*∗∗*^ *P* < 0.01 compared with baseline.

To examine whether the reduction of plasma proinflammatory cytokines by PIO treatment might be associated with downregulation of the mRNA expressions of proinflammatory cytokines in MNC, quantitative real-time PCR was used to calculate the mRNA expression. In the Con group, the mRNA expressions of IL-6, TNF-*α*, and MMP-9 at 12 weeks' follow-up were increased compared with the baseline (paired *t*-test, *P* = 0.009, 0.025, and 0.105, resp., Figures 2(a)–2(c)). Compared with the Con group, PIO treatment significantly reduced the mRNA expressions of IL-6, TNF-*α*, and MMP-9 (TWRMANOVA, *P* = 0.031, 0.012, and 0.007 resp., Figures 2(a)–2(c)), indicating that PIO attenuate DES-induced proinflammatory factors expression in MNC.

### 3.4. Pioglitazone Regulated the DES-Induced NF-*κ*B/PPAR-*γ* Imbalance In Vivo

To evaluate whether pioglitazone interacts with the proinflammatory transcription factor NF-*κ*B in MNC, we measured the nuclear NF-*κ*B DNA binding activity in MNCs (Figure 2(a)). As depicted in Figure 2(d), DES enhanced the DNA binding activity of NF-*κ*B (*P* = 0.023), which were attenuated by PIO (TWRMANOVA, *P* = 0.017).

Next, we detected the expressions of the p50 subunit and p65 subunit of NF-*κ*B in MNCs. As shown in Figure 2(e), PIO treatment significantly reduced DES-induced p50 expression (TWRMANOVA, *P* = 0.017) but did not alter p65 expression.

**Figure 3:**
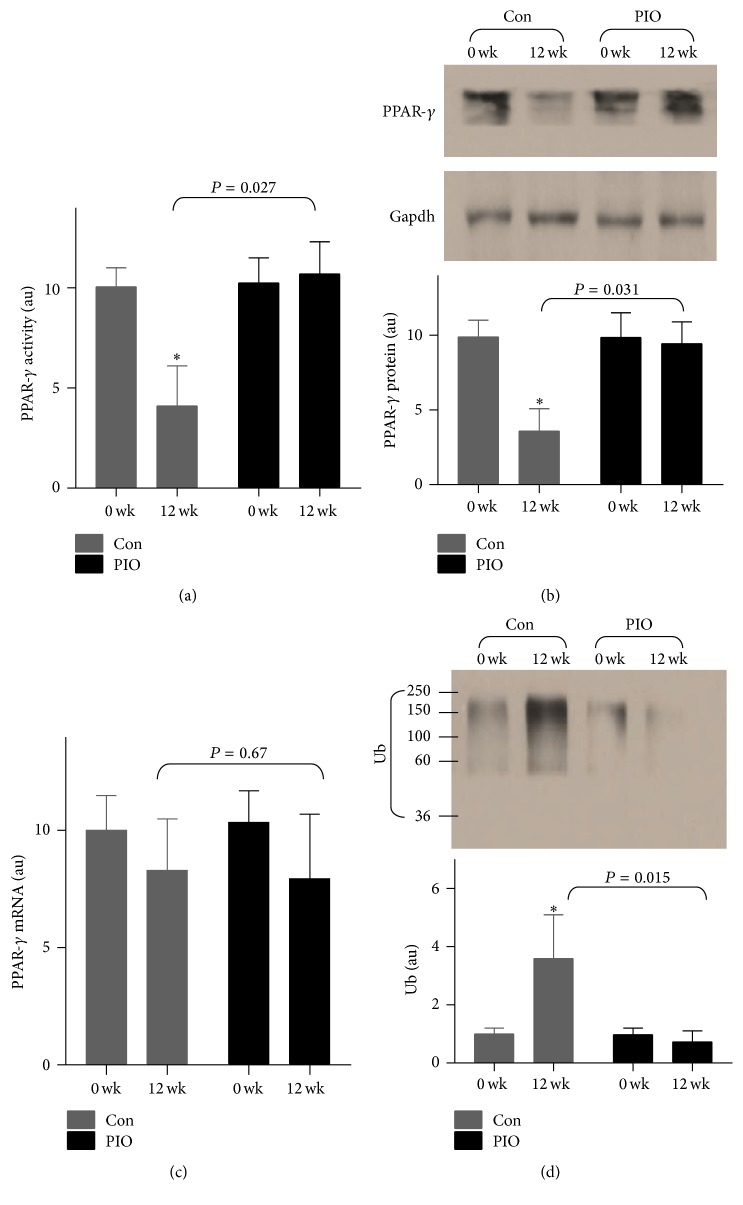
Pioglitazone blocked the DES-induced PPAR-*γ* ubiquitination and degradation in vivo. (a) PPAR-*γ* DNA binding activity was shown. (b) PPAR-*γ* protein was tested by Western blot. (c) The mRNA expression of PPAR-*γ*. (d) The ubiquitination of PPAR-*γ* was detected by coimmunoprecipitation assay. ^*∗*^ *P* < 0.05, compared with baseline.

Moreover, we found that PPAR-*γ* were dramatically decreased in the DES group (*P* = 0.038, Figure 3(a)), which were reversed by PIO treatment (TWRMANOVA, *P* = 0.027, Figure 3(a)), suggesting that pioglitazone regulated the DES-induced NF-*κ*B/PPAR-*γ* imbalance in vivo.

### 3.5. Pioglitazone Regulated the DES-Induced PPAR-*γ* Ubiquitination and Degradation

To refine the changes of protein and mRNA expressions of PPAR-*γ*, Western blot and quantitative real-time PCR were performed. As shown in Figure 3(b), DES markedly decreased PPAR-*γ* protein level, which were blocked by PIO treatment (TWRMANOVA, *P* = 0.031, Figure 3(b)). We also found that PIO did not alter PPAR-*γ* mRNA expression, suggesting that PIO may regulate the protein stability in vivo. Furthermore, we detected the ubiquitination of PPAR-*γ* by Western blot. As depicted in Figure 3(d), DES significantly induced PPAR-*γ* ubiquitination (*P* = 0.043), which was blocked by PIO, implying that pioglitazone regulated the DES-induced PPAR-*γ* ubiquitination and degradation in vivo.

## 4. Discussion

The present study clearly showed that the circulating inflammatory responses were increased after the implantation of DES in the CAD patients treated with optimal drug combinations, which were blocked by addition of oral pioglitazone. PIO sequentially acts through PPAR-*γ* activation, NF-*κ*B blockade, and inhibition of inflammatory cytokine expressions. These findings suggested that PIO may have a novel direct protective role in modulating the proinflammatory responses after coronary DES implantation in CAD patients, thus providing further optimizations of the drug therapy in those patients.

Vascular inflammation is recognized as the foundation mechanism of atherosclerosis, and proinflammatory mediators including IL-6, TNF-*α*, and MMP-9 play a pivotal role in atherosclerosis [18]. IL-6 and TNF-*α* are classic proinflammatory cytokines, which play key roles in vascular disease [19]. Excessive degradation and remodeling of the extracellular matrix, a promoter of the instability of plaques, are the major effect of matrix metalloproteinases (such as MMP-9) [20]. DESs left target vascular intima partially unendothelialized for a sustaining period [21], resulting in intensely inflammation-arousing vessel segments, which may facilitate the release of multiple proinflammatory factors into serum and then proinflammation.

It has been reported that PIO may exhibit several antiatherosclerotic effects through multiple mechanisms, including modulation of blood pressure, lipid concentrations, matrix remodeling activation of matrix proteases, and finally induction of inflammation [22]. In the present study, we failed to observe a reduction of blood pressure, blood glucose, insulin, or HbA1c levels in both groups. Firstly, the unchangeable blood pressure and other metabolic parameters may be explained as follows: first, the blood pressure of all subjects was already under control by optimal treatment and the patients included in this study were all nondiabetics. Moreover, it has been reported that PIO treatment had no significant effect on the level of total cholesterol, LDL, and HDL. The improved lipid profile and hs-CRP are mainly attributed to the standard drug therapy.

Herein, we found a novel mechanism that PIO enhance PPAR-*γ* binding activity though inhibiting its ubiquitination and degradation, which may play a key role in PPAR function in vivo [23].

Taken together, our study demonstrated that PIO treatment attenuated the proinflammatory state in circulating MNCs; and our results suggest that PIO treatment may sequentially act through PPAR-*γ* activation, blocking of NF-*κ*B activation, and inhibition of inflammatory cytokine expressions. These findings suggest that PIO may have a novel direct atheroprotective role by modulating the local and circulating proinflammatory responses in patients with coronary polymer-based drug-eluting stent implantation.

## Figures and Tables

**Table 1:** Baseline characteristics of the study population.

Parameters	Placebo (*n* = 14)	Pioglitazone (*n* = 14)	*P* value
Sex, M/F, *n*/*n*	11/3	12/2	1.00
Age, yr	55.5 ± 10.9	56.2 ± 10.4	0.74
Body mass index, kg/m^2^	23.7 ± 4.9	24.5 ± 2.9	0.58
Systolic BP, mm Hg	124 ± 19	121 ± 16	0.72
Diastolic BP, mm Hg	78 ± 10	76 ± 10	0.55
Smoking	9	12	0.39
Fasting glucose, mmol/L	5.28 ± 0.57	5.32 ± 0.37	0.86
HbA1c, %	5.42 ± 0.59	5.18 ± 0.49	0.25
Total cholesterol, mmol/L	3.78 ± 0.76	3.61 ± 0.45	0.48
Triglycerides, mmol/L	1.75 ± 1.27	1.81 ± 1.23	0.89
HDL cholesterol, mmol/L	0.90 ± 0.17	0.88 ± 0.20	0.74
LDL cholesterol, mmol/L	2.35 ± 0.67	2.15 ± 0.49	0.34
Gensini score	12.6 ± 8.8	10.9 ± 5.1	0.54
hsCRP, mg/L	4.0 (0.8, 11.8)	2.9 (1.3, 12.3)	0.58
Treatment after stenting			
Aspirin	14	14	1.00
Clopidogrel	14	14	1.00
Blocker	12	14	0.48
ACE inhibitors/ARBs	14	14	1.00
Statins	14	14	1.00

Data are reported as mean ± SD, median (interquartile range), or *n*. BP, blood pressure; hs-CRP, high sensitivity C-reactive protein; ACE, angiotensin-converting enzyme; ARB, angiotensin II type 1 receptor blocker.

**Table 2:** Metabolic and other parameters at baseline and after 12 weeks.

Parameters	Placebo controls		Pioglitazone	
	0 wk	12 wk	0 wk	12 wk
Body mass index, kg/m^2^	23.7 ± 4.9	23.3 ± 3.3	24.5 ± 2.9	24.7 ± 2.5
Weight, kg	65.4 ± 12.7	66.7 ± 12.7	68.8 ± 9.4	69.3 ± 8.3
Waist to hip ratio, %	89.1 ± 5.8	89.3 ± 5.6	89.9 ± 5.9	90.2 ± 5.5
Systolic BP, mm Hg	124 ± 19	126 ± 13	121 ± 16	121 ± 15
Diastolic BP, mm Hg	78 ± 10	77 ± 8	76 ± 10	73 ± 9
Fasting glucose, mmol/L	5.28 ± 0.57	5.30 ± 0.49	5.32 ± 0.37	5.31 ± 0.45
Fasting insulin, U/L	6.73 ± 3.98	6.10 ± 4.02	6.79 ± 4.44	5.21 ± 3.62
HbA1c, %	5.42 ± 0.59	5.72 ± 0.46	5.18 ± 0.49	5.79 ± 0.35
Total cholesterol, mmol/L	3.78 ± 0.76	3.12 ± 0.74^*∗∗*^	3.61 ± 0.45	3.01 ± 0.51^*∗∗*^
Triglycerides, mmol/L	1.75 ± 1.27	1.38 ± 0.91^*∗*^	1.81 ± 1.23	1.14 ± 0.39^*∗*^
HDL cholesterol, mmol/L	0.90 ± 0.17	1.16 ± 0.41^*∗*^	0.88 ± 0.20	1.27 ± 0.41^*∗∗*^
LDL cholesterol, mmol/L	2.35 ± 0.67	1.67 ± 0.40^*∗∗*^	2.15 ± 0.49	1.69 ± 0.40^*∗*^
hsCRP, mg/L	4.0 (0.9, 11.8)	2.2 (0.8, 6.8)^*∗∗*^	2.9 (1.3, 12.3)	1.0 (0.5, 3.8)^*∗∗*#^
NT-pro BNP, pg/mL	220 (79, 839)	231 (140, 664)	259 (73, 706)	260 (102, 703)
EF, %	61.3 ± 9.9	62.7 ± 9.5	60.4 ± 8.1	60.6 ± 7.4

*n* = 14 for each group. Values are mean ± SD or median (interquartile range).

^*∗*^ *P* < 0.05 and ^*∗∗*^ *P* < 0.01 compared with baseline, and ^#^ *P* < 0.01, compared with the placebo group.
