# Corrigendum to “Pioglitazone Attenuates Drug-Eluting Stent-Induced Proinflammatory State in Patients by Blocking Ubiquitination of PPAR”

**DOI:** 10.1155/2019/3298724

**Published:** 2019-03-03

**Authors:** Zhongxia Wang, Tao Zhang, Lizhe Sun, Ruifeng Li, Yuanyuan Wei, Xiaojuan Fan, Zuyi Yuan, Junhui Liu, Tao Chen

**Affiliations:** ^1^Department of Cardiology, First Affiliated Hospital of Xi'an Jiaotong University, Xi'an 710061, China; ^2^Department of Medicine, Gansu Provincial Hospital of TCM, Lanzhou 730050, China; ^3^Department of Cardiology, Xi'an Central Hospital, Xi'an 710003, China

In the article titled “Pioglitazone Attenuates Drug-Eluting Stent-Induced Proinflammatory State in Patients by Blocking Ubiquitination of PPAR” [1], there are additional methods that should be added to the article which are mainly about the methods for PPAR gamma binding and ubiquitination. The additional methods are shown below:

*2.8. Immunoblotting. *Protein levels of p65, p50, PPAR-*γ*, and Gapdh of MNC were detected by western blotting with Santa Cruz antibodies against the p65 subunit (sc-372), p50 subunit (sc-114), PPAR-*γ* (sc-7273), and Gapdh (SC-48166) as described previously [24,25]. Mononuclear cells were lysed in RIPA buffer (Cybrdi, Gaithersburg, MD, USA) that contained protease inhibitor (Roche Diagnostics, Indianapolis, IN, USA). Equal amounts of protein were loaded onto 4–12% Bis-Tris precast gels for electrophoresis and were electrotransferred onto a PVDF membrane (Roche Diagnostics). After blocking for 1 h at room temperature, membranes were sequentially incubated with primary Abs at 4°C overnight and secondary Abs at room temperature for 1 h. The protein signal was detected by chemiluminescence and all values were corrected by loading with Gapdh.

*2.9. Immunoprecipitation*. Immunoprecipitation assays were performed as described previously [25, 26]. Cell extracts were prepared by using modified RIPA buffer (Cell Signaling Technology). Cell lysates (300 *μ*g) were incubated with 1 *μ*g of PPAR Ab (sc-7273) at 4°C overnight and precipitated with protein G agarose beads (Santa Cruz) at 4°C for 2 h. Immunoprecipitated proteins were separated by SDS-PAGE and transferred onto a PVDF membrane. Membranes were then sequentially incubated with primary ubiquitination Ab (SC-8017) and secondary Abs. Bands were visualized by using an ECL system.

In addition, the below part should be added to the Discussion section after the third paragraph:

“The peroxisome proliferator-activated receptor-*γ* (PPAR-*γ*) is a member of the nuclear receptor superfamily of ligand-dependent transcription factors which regulate adipocyte differentiation, glucose homeostasis, and macrophage activation [27]. PPAR-*γ* ubiquitination and degradation have been found in adipocyte, and proteasome inhibitors inhibited the downregulation of PPAR-*γ*  [28]. Proinflammation cytokine was also found to induce PPAR-*γ* degradation [29]. Herein, we found a novel mechanism such that PIO enhances PPAR-*γ* binding activity though inhibiting its ubiquitination and degradation, which may be important for TZDs clinical use.”

Moreover, the references below should be included in the References list:

 
[24] T. Chen, X. Jin, B. H. Crawford et al., “Cardioprotection from oxidative stress in the newborn heart by activation of PPARg is mediated by catalase,”* Free Radical Biology & Medicine*, vol. 53, no. 2, pp. 208–215, 2012.
 
[25] C. Xie, T. Yagai, Y. Luo et al., “Activation of intestinal hypoxia-inducible factor 2*α* during obesity contributes to hepatic steatosis,”* Nature Medicine*, vol. 23, no. 11, pp. 1298–1308, 2017.
 
[26] Q. Zhao, D. Zhou, H. You et al., “IFN-*γ* aggravates neointimal hyperplasia by inducing endoplasmic reticulum stress and apoptosis in macrophages by promoting ubiquitin-dependent liver X receptor-*α* degradation,”* The FASEB Journal*, vol. 31, no. 12, pp. 5321-5331, 2017.
 
[27] L. Fajas, D. Auboeuf, E. Raspé et al., “The organization, promoter analysis, and expression of the human PPARgamma gene,”* The Journal of Biological Chemistry*, vol. 272, no. 30, pp.18779–89, 1997.
 
[28] S. Hauser, G. Adelmant, P. Sarraf, H. M. Wright, E. Mueller, B. M. Spiegelman, “Degradation of the peroxisome proliferator-activated receptor gamma is linked to ligand-dependent activation,”* The Journal of Biological Chemistry*, vol. 275, no. 24, pp. 18527–18533, 2000.
 
[29] K. J. Waite, Z. E. Floyd, P. Arbour-Reily, J. M. Stephens, “Interferon-gamma-induced regulation of peroxisome proliferator-activated receptor gamma and STATs in adipocytes,”* The Journal of Biological Chemistry,* vol. 276, no. 10, pp. 7062–7068, 2001.
